# Mesonephric-like adenocarcinoma of the ovary

**DOI:** 10.1186/s13048-024-01383-7

**Published:** 2024-03-05

**Authors:** Yun Yang, Mengru Zhao, Qiuchen Jia, Huimin Tang, Tingwei Xing, Yang Li, Bin Tang, Lin Xu, Weiwei Wei, Hong Zheng, Ruxia Shi, Bairong Xia, Jiming Chen

**Affiliations:** 1 Dalian Medical University, Dalian, 116000 PR China; 2 Department of Gynecology, Changzhou Second People’s Hospital Affiliated to Nanjing Medical University, Changzhou, 213003 PR China; 3 Department of Gynecological Oncology, Division of Life Sciences and Medicine, the First Affiliated Hospital of University of Science and Technology of China, University of Science and Technology of China, Hefei, 230031 PR China

**Keywords:** Mesonephric-like adenocarcinoma, Ovarian neoplasms, Treatment, Case reports, Immunohistochemistry

## Abstract

Mesonephric-like adenocarcinoma is a new class of rare subtypes of the female reproductive system. Its clinical symptoms are similar to other types of ovarian tumors. The diagnosis is based on pathological and immunohistochemical methods. The main treatment option is surgery combined with chemotherapy. Few cases have been reported at home and abroad. We reported a case of a 45-year-old woman with a cystic solid mass in the left adnexa. The postoperative pathological diagnosis was mesonephric-like adenocarcinoma of the left ovary and mature cystic teratoma (partial infiltration of the small intestine). This case had no specific clinical symptoms. Immunohistochemical findings showed positive results of GATA3, TTF1, CD10, ER, and PR. Paclitaxel and carboplatin chemotherapy were given after the operation. Currently, no specific criteria are available for diagnosis and treatment of the disease. This article aims to improve the understanding of clinicians in this disease and create a basis for clinical diagnosis and treatment.

## Introduction

Mesonephric-like adenocarcinoma (MLA) is a new and rare subtype of female reproductive system tumors. The origin of MLA has not been determined. Some scholars believe MLA originates from remnants of mesonephros adjacent to the ovary, while others suggest that it may originate from the Mullerian epithelium and then differentiate along the mesonephric pathway [1]. Its clinical syndromes are similar to other types of ovarian cancer. No symptoms were usually observed in the early stage, while the main symptoms in the late stage are abdominal distension, abdominal mass, ascites, and other gastrointestinal symptoms. Its morphology, immunohistochemistry, and molecular characteristics are similar to mesonephric adenocarcinoma. Postoperative pathology is the golden standard for the diagnosis of ovarian MLA [2]. Currently, surgery is the first choice of treatment, followed by adjuvant chemotherapy [3]. This article aims to improve the understanding of clinicians in this disease and create a basis for clinical diagnosis and treatment.

## Case presentation

**Fig. 1:**
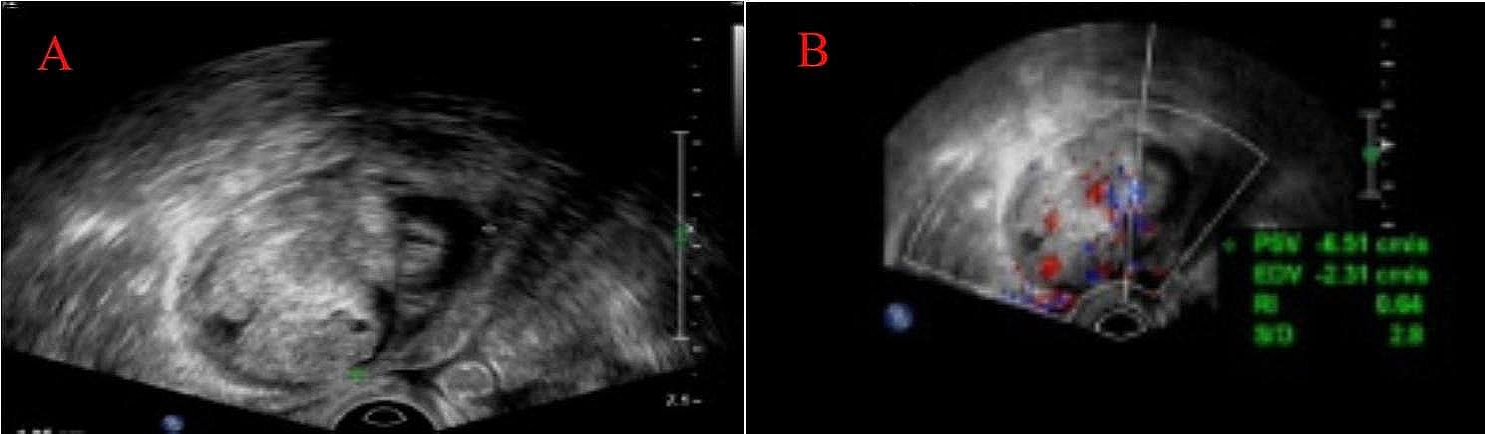
B-ultrasound image. Solid mixed pelvic echo area of 5.0 cm × 3.8 cm × 4.7 cm, with a clear boundary and regular morphology (**A**). CDFI: Rich blood flow signals can be detected within the arterial spectrum, with the arterial resistive index (RI) at 0.64. A free, nocturnal dark area was found in the pelvic cavity at a depth of about 1.7 cm (**B**)

**Fig. 2:**
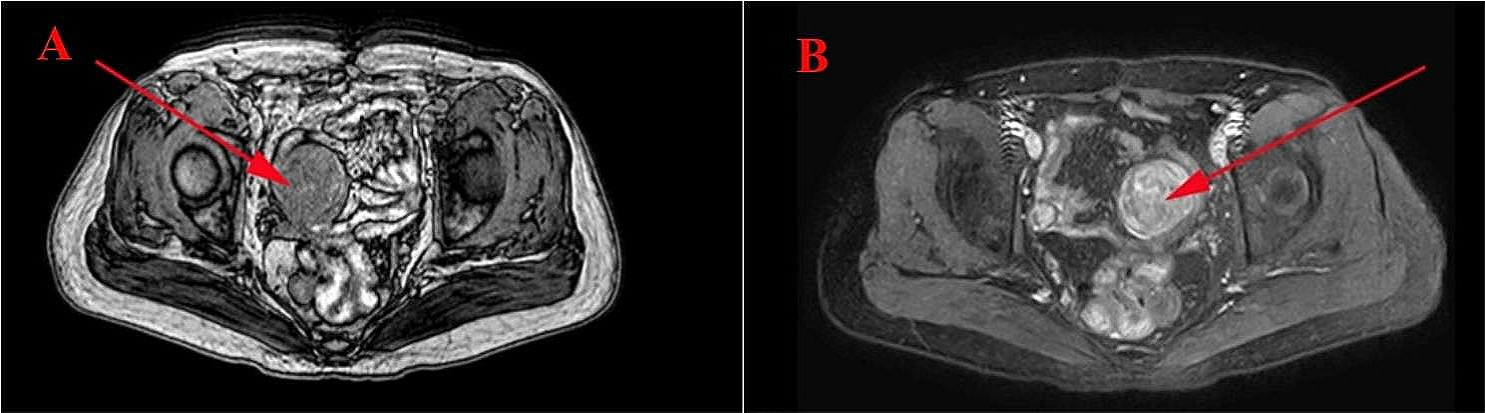
A cluster of abnormal signal lesions is seen in the left pelvic area, with a maximum cross-section of approximately 5.0 × 4.2 cm. The boundary is still clear, and the signal is uneven. It is iso-hypointense on T1WI and slightly hyperintense on T2WI. (**A**) DWI is hyperintense (**A**). Enhancement is uneven, and the boundary between the lesion and the left adnexa is unclear. (**B**). The red arrow indicates the location of the tumor

**Fig. 3:**
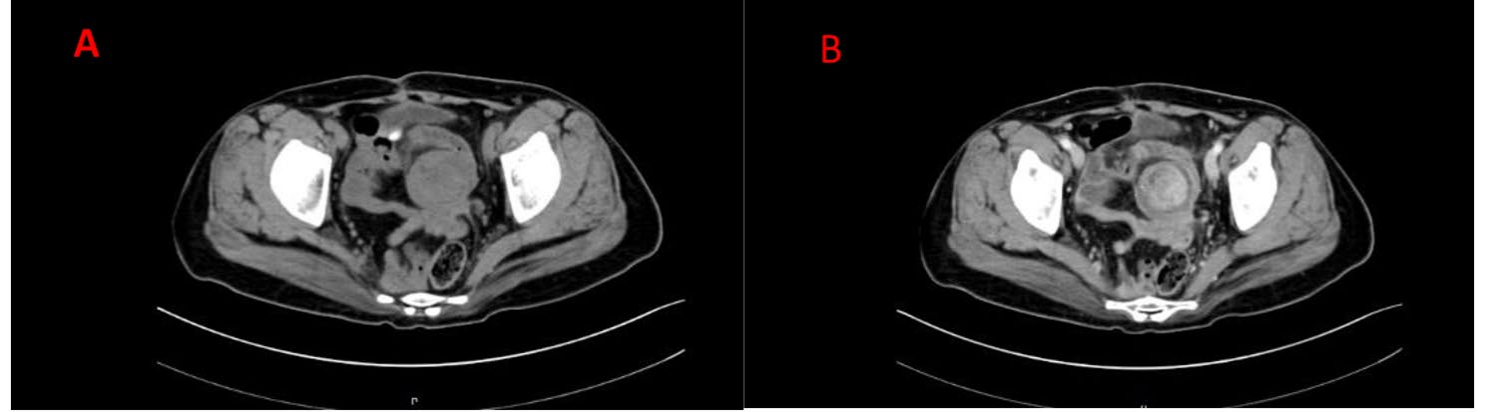
(**A**) A mass-like soft tissue density shadow is seen on the left side of the pelvis. The maximum cross-section is about 4.7 × 4.1 cm, with an unclear boundary of the lesion. (**B**) The enhancement showed obviously uneven strengthening. There was no obvious abnormal thickening in the intestinal walls of the small intestine, colon, and rectum, and the intestinal lumen had no obvious dilatation

**Fig. 4:**
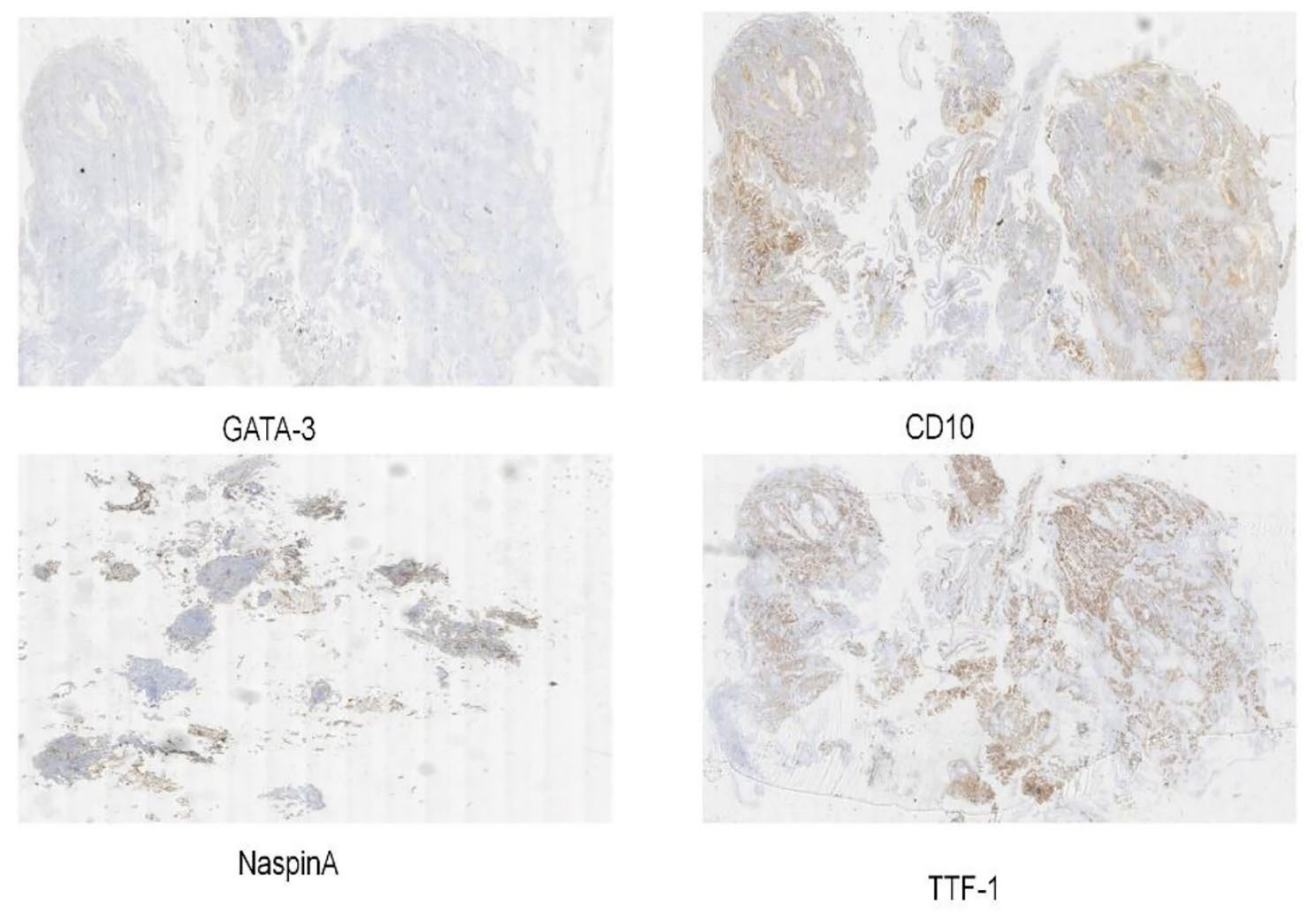
Immunohistochemistry: GATA-3 (small foci +), TTF-1 (+), CD10 (small foci +), NaspinA (-)

**Fig. 5:**
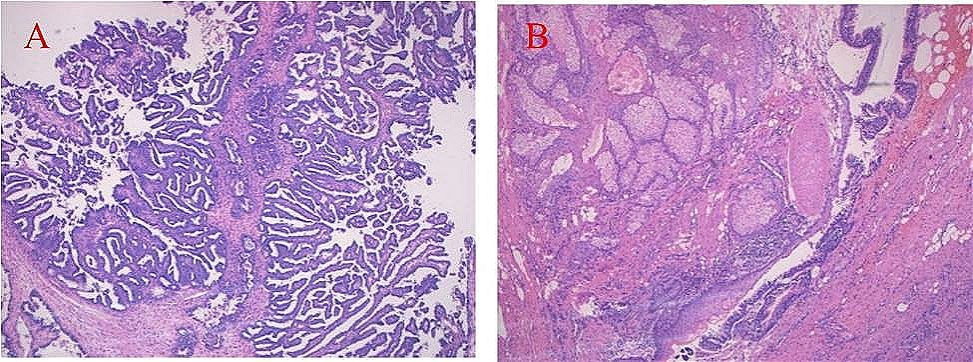
Invasive adenocarcinoma of the left ovary with a multitubular structure and eosinophilic secretions in the lumen (**A**), accompanied by histological and immunohistochemical tendencies of mesonephric-like adenocarcinoma and mature cystic teratoma (**B**)

**Fig. 6:**
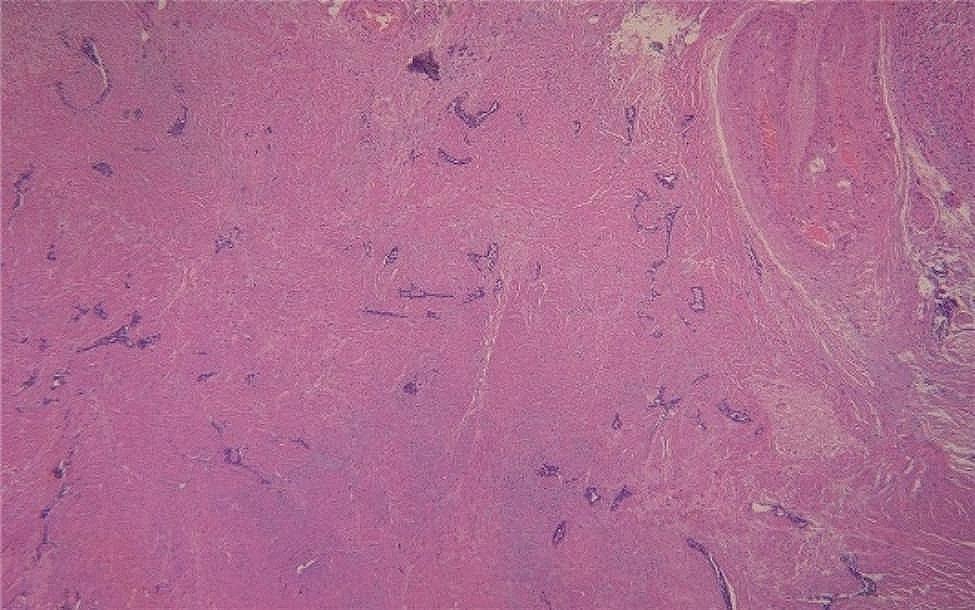
(Part of the small intestine) Focal infiltration was observed in the intrinsic muscular layer of the intestinal wall, and the upper and lower margins were negative

A 45-year-old female patient was admitted to the hospital on March 13, 2023, for an examination that revealed a left adnexal mass of more than 5 years’ duration and 1 month’s increase in size. The patient underwent an appendectomy 30 years ago, a cesarean Sect. 23 years ago with a lower uterine segment transverse incision, a laparoscopic total hysterectomy, right ovarian cyst removal surgery, bilateral salpingectomy, abnormal focus in the pelvic cavity, pelvic adhesion separation, and intestinal adhesion separation surgery (due to chocolate cyst on the right ovary) 5 years ago. The postoperative recovery was good. The patient was found during a physical examination 5 years ago to have a left adnexal mass measuring approximately 1 cm × 1 cm, which persisted without significant enlargement. The patient underwent a B-scan ultrasonography 1 month ago, showing a solid mixed pelvic echo area of 5.0 cm × 3.8 cm × 4.7 cm, with a clear boundary and regular morphology. CDFI: Rich blood flow signals can be detected within the arterial spectrum, with the arterial resistive index (RI) at 0.64. A free, nocturnal dark area was found in the pelvic cavity at a depth of about 1.7 cm [See Fig. 1]. The patient had a significantly larger left appendage mass than before and felt occasional symptoms of lower abdominal pain, nausea, and vomiting and was hospitalized. Examinations after hospitalization were as follows. Pelvic MRI (routine + enhanced): The left pelvic region had a mass of abnormal signal foci, with the largest cross-section being about 5.0 × 4.2 cm, clear lesion boundary, and not homogeneous signal. T1WI showed low iso-intensity signals, T2WI showed slightly high signal intensity, DWI showed high signal intensity, and the enhancement was unevenly strengthened. The boundary between the lesion and the left adnexa was not clear. It was considered a malignant tumor of left adnexal origin [See Fig. 2]. CT of the whole abdomen (routine + enhanced): A soft-tissue mass shadow was observed on the left side of the pelvis, with a maximum cross-section of about 4.7 × 4.1 cm. The lesion boundary was not clear. The enhancement showed obvious inhomogeneous strengthening. The intestinal walls of the small intestine, colon, and rectum did not show obvious abnormal thickening, and the intestinal lumen did not show obvious dilatation [See Fig. 3]. Tumor markers: CEA 2.0 ng/mL, AFP: 3.75 ng/mL, CA125 100.7 U/mL↑, CA199 266 U/mL↑, CA50: 203.76 U/mL, HEF: 114.3 pmol/L, SCC: 0.9 ng/mL, neuron-specific enolase 13.51ng/mL (all within the normal range). Based on the patient’s medical history and examination results, the preliminary diagnosis was a possible ovarian tumor. After a total hysterectomy, Laparoscopic surgery was performed under general anesthesia on March 15, 2023. During the surgery, the uterus and right adnexa were absent, no obvious ascites were seen in the pelvis, and extensive dense adhesions were observed between the sigmoid colon, small intestine, rectum, and its mesentery and both pelvic walls, pelvic floor, and vesico-peritoneal anticlinal folds. After the separation of membranous adhesions between small bowel loops, the left ovary was found to be enlarged in size at about 5 cm × 4 cm × 4 cm. Two small fat-like nodules were seen in the mesentery of the small intestine, with a diameter of about 0.3cn or 0.4 cm, and several hard nodules could be palpated in the pelvic peritoneum of the left pelvis (at the adhesion to the left ovary), with a diameter of about 0.3 to 0.5 cm. The surface of the right peritoneum, greater omentum, liver, and diaphragm was smooth. The left ovary was filled with cystic, solid tumor tissue, gray-white in color, with bad and brittle texture. Intraoperative rapid pathology: The left ovary tumor gland hyperplasia was active with cellular heterogeneous junctional changes. Part of the complex gland had a disorder with necrosis, part of the tumor glands had carcinoma, and the tumor tissue had a small amount of morphology and mild squamous epithelium, which should be further clarified through routine checks. It was decided to perform laparoscopic unilateral salpingo-oophorectomy, laparoscopic pelvic lymph node dissection, para-abdominal aortic lymph node dissection, omentectomy, partial resection of the small intestine, and resection of peritoneal lesions. Immunohistochemistry: GATA-3 (small foci +), TTF-1 (+), CD10 (small foci +), Vim (+), ER (partial +), PR (small foci +), HER2 (1+), Ki-67 (+, 30%), Pax-8 (+), Pax-2 (+), PTEN (+), B-catenin (plasma membrane +), MSH2 (+). MSH6 (+), MLH1 (+), PMS2 (+), p53 (+, wild-type), WT-1 (-), P16 (mottled+), NaspinA (-), D2-40 (-), MC (partially +), Calretinin (small foci+), TG (-) [See Fig. 4]. Postoperative pathology: Invasive adenocarcinoma of the left ovary, combined with histologic patterns and immunohistochemical predisposition to mesonephric-like adenocarcinoma (MLA); mature cystic teratoma [See Fig. 5]. (Part of the small intestine) Focal infiltration was observed in the intrinsic muscular layer of the intestinal wall, and the upper and lower margins were negative [See Fig. 6]. No malignant tumor cells were found in the ascites, and there was no lymph node metastasis. FIGO staging is IIIA2, The patient’s tumor markers gradually decreased after surgery, with CA125 at 37.69 U/mL and CA199 at 66.48 U/mL. Six courses of paclitaxel + carboplatin chemotherapy were proposed after surgery. No obvious contraindications to chemotherapy were found in the examinations. The patient started to receive paclitaxel (Lipitor) 210 mg + carboplatin 0.55 g chemotherapy from April 24, 2023, and the next chemotherapy was scheduled after 21 d. The patient is now in her fourth course of chemotherapy.

## Discussion

Based on a systematic literature search using the terms “mesonephric-like adenocarcinoma” and “ovary” in the PubMed, Wanfang, and CNKI databases, 19 mesonephric-like adenocarcinoma of the ovary cases were reported, as shown in Table 1.

**Table 1:** Review of the literature on mesonephric-like adenocarcinoma (MLA)

Case	Age	associated Findings	FIGO Stage	Treatment	Chemotherapy OR Radiation Y/N	Recurrence Y/N	Follow-up time M	Immunohistochemical	Molecular	Microhistology	Symptoms	Size	Number of cases
Chapel et al/ 2018 [4]	80	Low-grade serous carcinoma and serous borderline tumor	NA	total abdominal hysterectomy with bilateral salpingooophorectomy, omentectomy, and tumor debulking	Y,neoadjuvant chemotherapy with carboplatin and paclitaxel	N	Three M	diffusely positive for CK7, EMA, and PAX8 ,focally positive for p16, p53‘wildtype’ ,positive for GATA-3, TTF-1, inhibin,p63,CD10,negative for CK20,thyroglobulin,chromogranin, synaptophysin, calretinin, CDX2, parathyroid hormone,PR, WT1, ER	gains in chromosome 1q and 18p and losses in chromosomes 1p, 18q, and 22,mutations in the tumor suppressor genes BCOR or AMER1	predominantly small open and closed tubules, with foci of solid growth and single-cell invasion,Tumor cells were relatively monotonous with mild to moderate cytologic atypia and small nucleoli. Mitotic activity was low (1 to 2 mitotic figures per 10 hpf).	abdominal pain,pelvic mass	11.8*12.5*8.9 cm	1
McCluggage WG et/2020 [5]	61	Serous borderline tumors of both ovaries, low-grade serous carcinomas, and endometriosis	IIIA1	Hysterectomy, bilateral salpingo-oophorectomy, Douglas pouch peritonectomy, omentectomy, and bilateral pelvic lymphadenectomy, peritoneal washout, and cytoreductive surgery	Six cycles of adjuvant chemotherapy (carboplatin and paclitaxel)	NA	NA	PAX8 and CD10 were diffusely positive, GATA3 and TTF1 were focally positive, p53 was “wild type” immunoreactive, and WT1, ER, PR and thyroglobulin were negative	KRAS mutation	Intracavitary eosinophilic secretions, nuclear monomorphism, angulation, moderately atypical, with mitotic and focal necrotic areas	Pelvic pain, increased vaginal discharge, pelvic mass	The maximum size is 6 cm	1
McFarland M/2016 [2]	42 ~ 72,Average age 60	There were 3 cases of endometriosis in the same ovary	IA(3Case),IB(1Case),IIIC(1Case)	NA	NA	Still alive at the time of 12 months	18 months	ER and PR were completely negative, CK 7 and PAX 8 were positive	NA	Tubular, glandular, papillary, falliform and solid structures. The tubules had luminal acidophilic material. The epithelial cells showed moderate nuclear atypia. The nuclei were atypical, clear or cystic and angulated.	NA	The size ranges from 4 to 32 cm	5
Pors, J et/2018 [10]	67	N	IC	NA	NA	NA	NA	ER and reticulin were completely negative, GATA3, TTF1, PAX8 and CD10 were positive	NA	NA	NA	NA	1
Dundr P,et,2020 [7]	61	Serous borderline tumor	IV	Hysterectomy with bilateral salpingo-oophorectomy, resection of hepatic metastases, greater omentum, and appendix	Five cycles of neoadjuvant chemotherapy were followed by adjuvant chemotherapy for 12 months	N	11 months	PAX8,TTF1和 GATA3的阳性	KRAS and PIK3CA somatic mutations.MYCN mutation, loss of exon 9-10 in CHEK2 gene	Tubular, cystic and solid structures. Dense eosinophilic material.The tumor cells were medium in size, dominated by vesicular nuclei and small nucleoli, with 4 /10 HPF	NA	NA	1
Nilforoushan N/2023 [8]	58 and 70 years old	Case 1: Left ovarian serous borderline tumor, right ovarian serous borderline tumor and low grade serous carcinoma. Case 2: Serous borderline tumor of the left ovary	IVB	Total hysterectomy and bilateral salpingo-oophorectomy and omentectomy	NA	NA	NA	GATA3 was diffusely positive, WT-1, ER and PR were negative, p16 was focally and patchy positive, p53 was wild type, Napsin A, TTF-1, CD 10 and PD-L1 were negative	KRAS G12V mutation	NA	NA	NA	2
McCluggage WG et/2018 [17]	50-77 years old, average age 66.5 years old	One case of borderline endometrioid adenoma, one case of endometriosis, mixed serous and mucinous cystadenoma, and one case of serous cystadenoma	NA	NA	NA	NA	NA	NA	NA	NA	NA	NA	4
seay/2020 [18]	67	Endometriosis	IA	Exploratory laparotomy, total abdominal hysterectomy, pelvic lymphadenectomy, inferior omental resection, and adhesiolysis were performed	Chemotherapy combined with carboplatin and paclitaxel was given 18 months after surgery for tumor metastasis	Y	NA	CK7 and PAX-8 were strongly positive.HNF 1B, TTF 1, CD 10 and p16 were focally positive, while ER, PR, WT-1 and calretinin were negative.	ATM, PALB 2 gene variation	Tubular, glandular, papillary, chordlike and solid.Cytoplasmic sparseness, nuclear overlap, moderately atypical nuclei with vesicle changes, unmarked nucleolus, and increased mitotic activity	Abdominal pain, polyuria	11 cm	1
樊静华/2022 [20]	60	N	Ib	Total hysterectomy, bilateral adnexa and omentum resection and pelvic adhesiolysis	NA	N	12 months	TTF1, CD10, KatA-3, PAX8, CK7, p16, CK20, CA125, Vimintin, Napsin A were all or partially positive, p53 was expressed in wild type, Ki-67 index was about 30%,ER, PR, WT-1, Calritinin, Ber-EP4 were negative	NA	Papillary, glandular, tubular and sieve shaped arrangement, eosinophilic secretions can be seen in some lumen, the nucleus is round or oval, the nucleolus is not obvious, no mitotic image	Nausea, vomiting, and abdominal distention	7.9 cm×7.3 cm×4.7 cm	1
张方圆/2022 [19]	61	N	IVB	Transabdominal cytoreductive surgery (including bilateral appendectomy, partial colectomy, partial rectal resection, colorectal anastomosis, and omentectomy) was performed	5 courses of paclitaxel + carboplatin chemotherapy	N	13 months	Negative for ER, PR, WT1 and Desmin, positive for PMS2, SALL-4, MLH1, MSH6, MSH2, H-Caldesmon, SMA, AFP and NapsinA, positive for CD10, EMA, CK7, HNF1-β(focally +), Vimentin and CK, GATA3, P16, TTF-1(partial +), α-inhibin(scattered +), P53(weak positive), Ki67(positive 60%)	NA	There were multiple tubular structures with eosinophilic secretions in the lumina	Abdominal distention	12.9 cm×12.2 cm	1
张世凯/2022 [21]	67	Borderline seromucinous neoplasm, endometriosis	IC	Hysterectomy, bilateral adnexa, omentum, appendectomy and pelvic lymph node dissection	Paclitaxel 270 mg and carboplatin 600 mg were given once	NA	4 months	PR was weakly positive, CK20 was scattered positive, Ki-67 proliferation index was 40%,PAX-8 (staining results were unclear), P16, P53 and CK7 were partially positive, CK(AE1/AE3), TTF-1 and GATA3 were positive.ER, CDX2, WT1, calretinin, alpha inhibin, vimentin, HNF1β, NapsinA and P504s were all negative	NA	Papillary, ductal, solid, less cytoplasm, eosinophilic colloid - like secretion in the lumen.Covering epithelium in clusters of hyperplasia.	Pelvic mass and frequent urination	12.2 cm×9.2 cm×9.9 cm	1
present case/2023	45	endometriosis	IIB	Laparoscopic unilateral salpingo-oophorectomy, laparoscopic pelvic lymph node dissection, para-abdominal aortic lymph node dissection, salpingo-oophorectomy, partial resection of small bowel, resection of peritoneal lesions	Paclitaxel (Lioxine) 210 mg+ carboplatin 550 mg chemotherapy for 6 courses	N	4 months	GATA-3, PR, Vim, TTF-, Pax-8, Pax-2, PTEN, B-catenin, MSH2, MSH6, MLH1, PMS2 and wild-type p53 were all positive.NaspinA, D2-40, WT-1, TG were negative,MC and ER were partially positive, Calretinin and CD10 foci were positive, P16(mottled +), Ki-67(+, 30%), HER2(1+).	NA	Multiple tubular structures, eosinophilic secretions found in the cavity	Abdominal pain and pelvic mass	5.0 cm × 3.8 cm × 4.7 cm	

### Pathogenesis

Mesonephric-like adenocarcinoma and mesonephric-duct adenocarcinoma are rare malignancies of the female reproductive system. Current studies suggest that mesonephric-duct adenocarcinoma is of mesonephric origin, often occurring in the cervix and vagina, and is associated with mesonephric ductal remnants and mesonephric ductal hyperplasia. Mesonephric ductal remnants may be seen around the tumor tissue. Mesonephric-like adenocarcinoma, a new class of rare subtypes included in the WHO (2020) Classification of Tumors of the Female Reproductive System, does not have mesonephric ductal remnants around the tumor tissue but has histologic, immunohistochemical, and molecular features similar to those of mesonephric-duct adenocarcinoma [1]. The histologic origin of mesonephric-like adenocarcinoma has been controversial. It has been suggested that it may originate from the epithelium of the Müllerian duct and then differentiate along the mesonephric pathway [2]. Among the 39 cases of ovarian mesonephric-like adenocarcinoma statistically analyzed by Deolet E [3] et al., 16 were associated with the epithelium of the Müllerian duct. Yano et al., Dundr et al., McCluggage et al., and Chapel et al. found that mesonephric-like adenocarcinomas and endometrial carcinomas, plasmacytoid junctional tumors, and low-grade plasmacytoid carcinomas of the ovary share common mutations in the KRAS and NRAS genes, which also supports the idea that mesonephric-like adenocarcinoma (MLA) is of the Mullerian origin [4–7]. In this case, the patient had a history of endometriosis (EMS), and the coexistence of ovarian-like adenocarcinoma and mature cystic teratoma of the ovary in this pathologic tissue suggested that the mesonephric-like adenocarcinoma in the ovary may originate in the Mullerian duct.

### Clinical manifestations and pathology

Mesonephric-like adenocarcinoma (MLA) of the ovary is extremely rare clinically, and there are few related reports at home and abroad. Its clinical manifestations are not significantly different from other types of ovarian cancers, often asymptomatic in the early stage, and chronic pelvic pain and abnormal uterine bleeding, postmenopausal irregular bleeding, abdominal distention, or asymptomatic in the late stage. The confirmation of diagnosis depends on pathological and immunohistochemical methods [8]. Histologically, mesonephric-like adenocarcinoma may show different patterns, such as reticular, papillary, tubular, gonadal, glandular, glomeruloid, fusiform, and sieve-like, and the same tumor often has a mixture of two or more growth modalities. Eosinophilic colloid-like material is commonly found in the lumen of the tubules [9–11]. Cytologically, tumor cells are flat, cuboidal, and columnar, mildly to moderately heterogeneous, while highly heterogeneous is uncommon. The cytoplasm is eosinophilic; nuclei are flat or ovoid, with vesicular nuclei, nuclear grooves, and nuclear overlap visible, without squamous, ciliated, and mucinous chemotaxis, and there is no mesonephric remnant in the periphery [12]. Immunohistochemistry is important for its diagnosis. Mesonephric-like adenocarcinoma is often positive for calretinin, GATA-3, CD10, and TTF-1, sometimes positive for P53, and negative for ER and PR. PR is a more reliable negative marker compared with ER [13]. Studies have shown that GATA3 and/or thyroid transcription factor 1 (TTF1) are considered as its markers [3]. Molecularly, G12V, and G12D are the most common KRAS mutations in mesonephric-like adenocarcinoma, and concurrent ARID1A and PIK3CA mutations are relatively common, with 1q copy number gain being the most common [2, 4, 14, 15]. Some of them also had a 1p loss. p53 expression adopts a wild-type pattern, p16 is patchy and WT1 is negative. However, no PTEN mutations/deletions were observed, microsatellite was unstable, which is different from the mesonephric-duct adenocarcinoma [16–18]. Studies have shown that the combined testing of CA125, CA199, and CEA in patients with ovarian malignant tumors can improve the accuracy of early diagnosis of ovarian cancer. In this case, the patient demonstrated preoperative elevation of CA125 and CA199 and postoperative declines, which can help predict the disease progression and prognosis evaluation [19–21].

### Treatment

Studies have shown that mesonephric-like adenocarcinoma is highly invasive and can recur and metastasize at an early stage. 39% of patients with mesonephric-like adenocarcinoma of the ovary were at stages II-IV at the time of diagnosis, and 43% of patients had lymphatic metastases. Tumors are more likely to metastasize when they have a diameter of > 4 cm, unclear tumor boundary, relatively high clinical stage (III-IV), large areas of necrosis, high nuclear fission index (> 10/10HPF), and lymphovascular invasion [12]. There is currently no uniformly recommended surgical method, and the general approach is total hysterectomy with bilateral adnexectomy, omentectomy, and pelvic lymphadenectomy. After surgery, chemotherapy, radiotherapy, or combined radiotherapy is used. Paclitaxel combined with carboplatin is generally recommended as the first-line chemotherapy regimen [22]. Inhibition of the RAS/MAPK pathway can also be a therapeutic modality when the mesonephric-like adenocarcinoma is combined with KRAS/NRAS mutations [23]. After standard treatment, 42% of patients may still experience a recurrence, and the most common sites of recurrence are the lung, omentum majus, liver, ossa pubis, perihepatic, mesentery, and peritoneum regions [22].

## Conclusion

Mesonephric-like adenocarcinoma of the ovary is a rare gynecologic malignancy that is easy to be misdiagnosed and underdiagnosed clinically, and the current diagnosis mainly relies on immunohistochemistry of postoperative pathology. Due to its rarity and non-standardized treatment yet, we expect the discovery of specific factors and indicators that can help with the diagnosis of this disease, which facilitates the development of clinical diagnostic criteria and treatment protocols for this disease. Ultimately, the disease can be detected and treated at an early stage to benefit the patients.
